# A Phylogeographic Survey of the Pygmy Mouse *Mus minutoides* in South Africa: Taxonomic and Karyotypic Inference from Cytochrome *b* Sequences of Museum Specimens

**DOI:** 10.1371/journal.pone.0098499

**Published:** 2014-06-06

**Authors:** Pascale Chevret, Terence J. Robinson, Julie Perez, Frédéric Veyrunes, Janice Britton-Davidian

**Affiliations:** 1 Laboratoire de Biométrie et Biologie Evolutive, UMR CNRS 5558, Université Lyon 1, Villeurbanne, France; 2 Evolutionary Genomics Group, Department of Botany and Zoology, University of Stellenbosch, Stellenbosch, South Africa; 3 Institut des Sciences de l’Evolution de Montpellier, UMR CNRS 5554, Université Montpellier 2, Montpellier, France; Tel-Aviv University, Israel

## Abstract

The African pygmy mice (*Mus*, subgenus *Nannomys*) are a group of small-sized rodents that occur widely throughout sub-Saharan Africa. Chromosomal diversity within this group is extensive and numerous studies have shown the karyotype to be a useful taxonomic marker. This is pertinent to *Mus minutoides* populations in South Africa where two different cytotypes (2n = 34, 2n = 18) and a modification of the sex determination system (due to the presence of a Y chromosome in some females) have been recorded. This chromosomal diversity is mirrored by mitochondrial DNA sequences that unambiguously discriminate among the various pygmy mouse species and, importantly, the different *M*. *minutoides* cytotypes. However, the geographic delimitation and taxonomy of pygmy mice populations in South Africa is poorly understood. To address this, tissue samples of *M*. *minutoides* were taken and analysed from specimens housed in six South African museum collections. Partial cytochrome b sequences (400 pb) were successfully amplified from 44% of the 154 samples processed. Two species were identified: *M. indutus* and *M. minutoides*. The sequences of the *M. indutus* samples provided two unexpected features: i) nuclear copies of the cytochrome b gene were detected in many specimens, and ii) the range of this species was found to extend considerably further south than is presently understood. The phylogenetic analysis of the *M. minutoides* samples revealed two well-supported clades: a Southern clade which included the two chromosomal groups previously identified in South Africa, and an Eastern clade that extended from Eastern Africa into South Africa. Congruent molecular phylogenetic and chromosomal datasets permitted the tentative chromosomal assignments of museum specimens within the different clades as well as the correction of misidentified museum specimens.

## Introduction

The African pygmy mice (subgenus *Nannomys*) represent an early African offshoot of the *Mus* lineage and are characterized by their overall small size (<10 g). Colonization of Africa brought about an extensive diversification of this monophyletic subgenus [1]–[3] which comprises 18 species distributed south of the Sahara [4]. Preliminary molecular data have shown that some of the *Nannomys* species are highly divergent, although they are often difficult to discriminate on morphological grounds owing to geographic variability and the lack of unambiguous diagnostic characters [2], [4], [5]. On the contrary, chromosomal characters have been useful taxonomic markers, and cytogenetic investigations have uncovered extensive karyotypic evolution within this group [6]. A case in point is *Mus minutoides* which shows three noteworthy features. First, this species has the most widespread distribution of the recognized taxa extending as it does throughout most of sub-Saharan Africa [7]. Second, phylogenetic analyses highlighted the existence of at least three well-supported clades within *M. minutoides*: Western Africa, West-Central Africa and East/South Africa [3], [7], [8] and these intraspecific relationships are in agreement with an extensive chromosomal diversity. In the East/South African clade for example, in addition to the centric fusion between the sex chromosomes and autosome 1, two divergent chromosomal groups (2n = 34, 2n = 18) exist that differ in the number of autosomal fusions present in each [9]. The concordant patterns illustrated by chromosomal and molecular markers [2], [9] suggests that the accumulation of chromosomal rearrangements may have played a role in accelerating the genetic divergence between the taxa. Third, *M. minutoides* is one of the very few species of mammals that presents an atypical sex chromosome system–it is noteworthy for a high proportion of sex-reversed XY females [10]. The existence of XY females was first detected in South African specimens and subsequently confirmed in West African populations suggesting that the mutation likely occurred at the onset of the diversification of the lineage [11]. In summary, this species has undergone a remarkable karyotypic evolution that is paralleled by a high level of genetic structure making it a useful model for studying chromosomal evolution and speciation processes in general, and in small mammals in particular.

African pygmy mice are, however, notorious for their low trapping success and *M. minutoides* is no exception. This has hindered advances in studying their taxonomic and chromosomal diversity since their collection is time-consuming, expensive and largely serendipitous. Access to museum types and specimens offers an unprecedented opportunity to resolve taxonomic questions and engage in long-term biodiversity studies. Here, we report the outcome of a museum-based phylogeographic survey of *M. minutoides* throughout South Africa that highlights the usefulness of this approach for investigations of rare or difficult to sample taxa.

## Materials and Methods

### Material

Tissue samples were taken from imperfectly cleaned skulls or dried skins of 287 specimens housed in the small mammal collections of six South African Museums (Table 1). Although the *Mus minutoides* collections were our main interest, tissues from *M. bufo*, *M. neavei* and *M. musculoides* specimens were included when available. All samples were stored dry in eppendorf tubes and a subset of these (154) were processed in the ‘Degraded DNA facility’ in Montpellier, France (dedicated to processing low quality/quantity DNA tissue samples). Alcohol-preserved tissue samples were available for 19 individuals in the Durban and Iziko Museum collections; the DNA from these samples was extracted in a separate room to avoid contamination. Additional ethanol-preserved tissue samples of four wild-caught *M. minutoides* (1 specimen) and *M. indutus* (3 specimens) were included in the analysis (Table S1).

**Table 1:** List of the sampled museums.

Museum	City	Code	Samples (cytb)	Success rate (%)
Amathole Museum	King Williamstown	KM	44 (8)	18
National Museum	Bloemfontein	NMB	16 (14)	88
Durban Natural Science Museum	Durban	DM	20 (8)	40
Iziko Museum	Cape Town	ZM	17 (5)	29
McGregor Museum	Kimberley	mmK/m/	14 (9)	64
Transvaal Museum	Pretoria	TM	43 (24)	56
All			154 (68)	44

Number of pygmy mouse samples (skin or skull) tested for each of the six museums. Those that produced pygmy mouse cytb sequences are indicated in brackets.

### Methods

**Figure 1:**
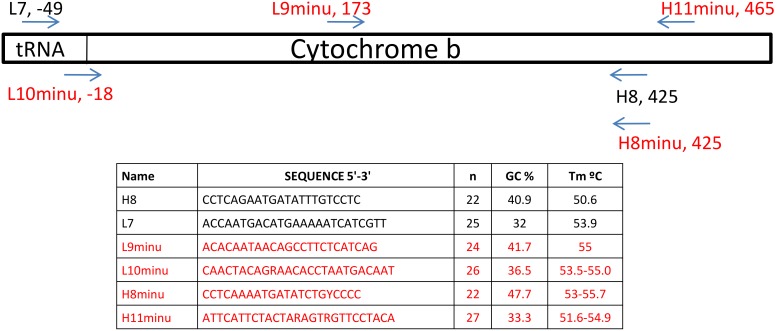
A schematic showing the respective positions of the primers used in this study. New primers defined specifically for use in the investigation are indicated in red.

Mitochondrial DNA analysis of degraded tissue is relatively straightforward provided appropriate controls and precautions are taken [12]–[14]. DNA was extracted using the DNEasy Blood and Tissue kit (Qiagen) following the manufacturer’s instructions, with a final elution of 100 ml of AE buffer. Museum samples were extracted in small batches (n = 7) and a negative control was included in each batch to monitor possible contaminations. Each batch included samples from different museums and localities. A fragment of the mitochondrial cytochrome b gene (cytb) (ca 400 bp) was first amplified using the primers L7 [15] and H8 [2]. PCR amplifications were performed in 25 µL reaction volumes containing 2.5 units of Perkin Elmer Gold Taq polymerase (Applied Biosystems), 2 mM MgCl2, 0.5 µM of each primer, 0.25 mM of dNTP, and 2 µl of sample extraction. For each PCR, the negative controls of each extraction batch and a PCR blank were included. The cycling conditions were: denaturation at 94°C for 5 min followed by 55 cycles at 94°C for 45 s, 50°C for 45 s and 72°C for 1 min, with a final extension at 72°C for 5 min. In order to check for the presence of contaminant DNA, the first PCR products were cloned using the TOPO TA Cloning Kit according to the manufacturer’s instructions (Invitrogen). Eight to ten positive clones per specimen were sequenced by Cogenics France (Grenoble, France). We used MEGA 5.1 [16] to edit the raw sequences and to build a consensus sequence for each sample. As these first steps sometimes led to the amplification of contaminant DNA, we designed primers specific to the subgenus *Nannomys* and/or *M. minutoides* based on the alignment of cytb sequences of several *Mus* species. These new primers and their positions are indicated in Figure 1; they are all located within the first 500 bases of the cytb gene, and were used in different combinations. Some samples (∼50%) were independently amplified twice to check the authenticity of the sequences. PCR products were sequenced in both directions by Macrogen Europe Laboratory (Amsterdam, The Netherlands).

The PCR products of eight specimens yielded two sequences using two different sets of primers; these were cloned and sequenced as described above. Comparison with the sequences from the ethanol-preserved tissues of the same species allowed us to determine which amplicons were from the mitochochondrial cytb gene and which were nuclear copies (*numts*).

For the ethanol-preserved tissues, the complete mitochondrial cytb gene was amplified as described in [2]. In instances where amplification failed (8 samples), a shorter fragment was sequenced as described above. The complete cytb sequences of the three wild-caught *M. indutus* specimens presented positions with double peaks. PCR products were cloned and the sequences (9–14 per specimen) were compared to those of the museum samples.

The new cytb sequences are deposited in the EMBL databank under accession numbers HG934890 to HG934979.

### Phylogenetic Analysis

A phylogenetic analysis was performed using the cytb sequences of the museum, wild-caught specimens as well as available sequences of *M. minutoides*; we also included sequences from several closely related species (*M. musculoides, M. indutus, M. mattheyi* and *M. haussa;* Table S2), the complete data set comprise 139 sequences (File S1). The best-fit model of sequence evolution was selected based on the corrected AIC score implemented in jmodeltest v2.1 [17]. The molecular phylogeny was reconstructed by Maximum Likelihood (ML) with RAxML v8.0 [18] and Bayesian analyses were performed using MrBayes v3.2.2 [19] under the model selected by jmodeltest (GTR + I + G) and three partitions corresponding to each codon position. The robustness of the nodes was estimated by bootstrap (BP) (1000 pseudoreplicates, starting tree: best tree obtained from 200 randomized MP starting tree) with RAxML and Posterior Probability (PP) using MrBayes - all parameters except topology are unlinked. Two simultaneous independent runs with 4 chains and 20 million generations each, were performed with one tree sampled every 500 generations, the burn-in of 5000 trees was determined with Tracer v1.6 [20], and we checked that the average SD of split frequencies remained <0.01 after the burn-in threshold. Average genetic divergence between and within each clade was evaluated under the Kimura 2-parameter distance model of nucleotide substitution using MEGA 5 [16].

## Results

### Molecular Analysis of Museum Specimens

A total of 68 cytb sequences of pygmy mice were obtained from the museum samples (skull or dried skin) yielding a mean extraction and amplification success rate of 44%. There was a large variation among the collections (18–88%; see Table 1). A small percentage (2%) of the extractions led to poor quality sequences that could not be used in the phylogenetic analysis. Among the initial extractions, 17% yielded contaminant (mostly human) DNA sequences–this proportion was considerably reduced when primers that specifically targeted pygmy mouse sequences were used. Interestingly, even relatively old samples (>50 years old, Fig. S1) provided good quality sequences. Unfortunately, none of the other species of pygmy mice yielded PCR products or uncontaminated DNA. The 23 ethanol-preserved samples (museum and wild-caught specimens) were all successfully amplified and sequenced. Among the museum specimens previously labelled as *M. minutoides*, 33% were found to be incorrectly identified and could be unambigously assigned to *M. indutus* on the basis of their cytb sequence.

### *Numt* Amplification

Irrespective of the tissue origin, some samples produced two distinct sequences among the different clones or between two PCRs performed with different sets of primers. This co-amplification may have several origins: it may result from a DNA contamination, or from the presence of a nuclear copy of the mitochondrial cytb (*numt*). Several observations argue against a contaminant origin for this sequence variation: i) the co-amplification occurred almost exclusively in specimens identified as *M*. *indutus*, ii) these samples were processed in different extraction batches, iii) the samples came from different museums, and more importantly, iv) the two sets of sequences were also present in the ethanol-preserved samples that were extracted in different laboratories.

**Figure 2:**
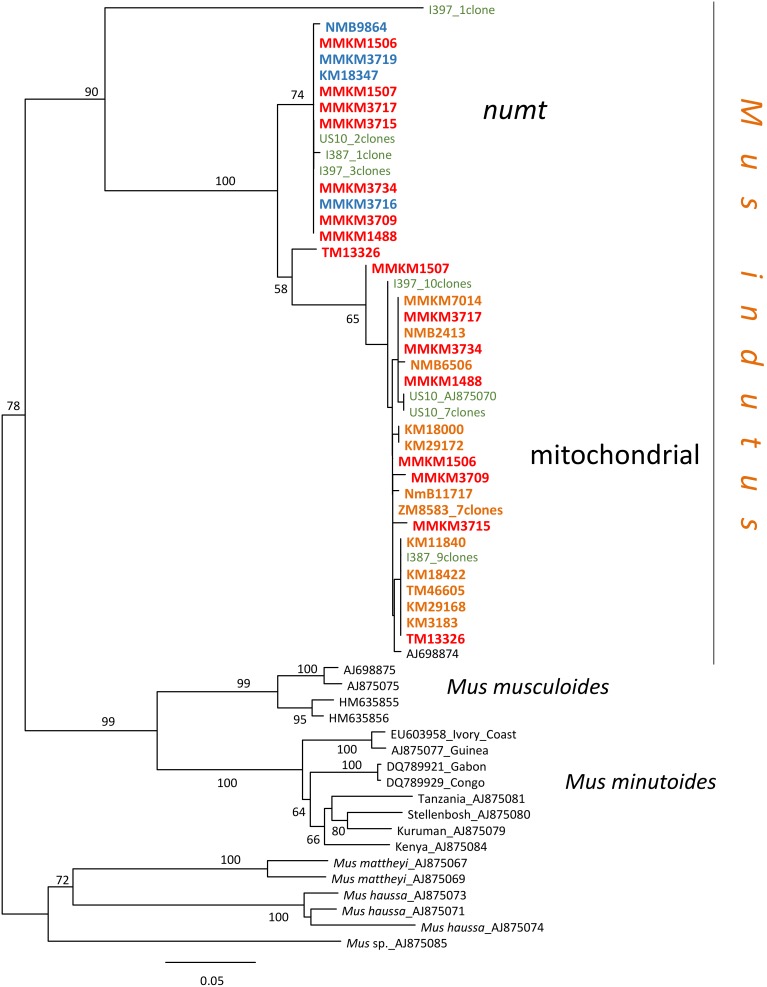
Maximum likelihood phylogeny of the *numt* and mitochondrial cytb sequences of *Mus indutus*. For each sample, the accession or collection number is indicated. The numbers in bold correspond to museum specimens; they are in red when two sets of sequences were obtained, in blue when only *numt* copies were sequenced and in orange when only mitochondrial copies were present. The three fresh tissue samples are in green. The number of clones selected for sequencing is shown. Boostrap supports above 50% are indicated.

PCR products of the complete cytb obtained from the three wild-caught *M. indutus* samples (I387, I397 and US10; Table S1) were cloned; 10, 14 and 9 clones were sequenced respectively. In all cases, two sets of sequences were retrieved. The two consensus sequences for the three wild-caught *M. indutus* samples were aligned with other *Nannomys* species (Fig. S2a) and translated into the protein (Fig. S2b). In these specimens, one sequence was always more abundant in the cloned sequences: 7 of the 9 clones for US10, 9 of the 10 clones for I387 and 10 of the 14 clones for I397. Most of the predicted proteins of these sequences appeared functional since there was no evidence of deletions or nucleotide substitutions that would result in frameshift or stop-codon mutations (Fig. S2a and b). Among the less abundant sequences one had lost the ATG codon in the first position (Fig. S2a), and one had a 7 bp indel (Fig. S2a) and several stop-codons (Fig. S2b). These minor sequences also presented an excess of non-synonymous substitutions compared to what is observed within and between other species of *Nannomys* (Fig. S2b). Based on these data, we considered that the less frequent sequences correspond to nuclear copies of the cytb. A phylogeny using these *numt* sequences, including those present in the *M. indutus* museum specimens, was reconstructed with RAxML v8.0 (Fig. 2). Among these samples, four specimens yielded only *numt* sequences, 12 only mitochondrial sequences, whereas two sets of sequences were retrieved for eight others (Fig. 2; Table S1). The *numt* sequences were not included in the final phylogenetic analysis.

### Molecular Phylogeny

**Figure 3:**
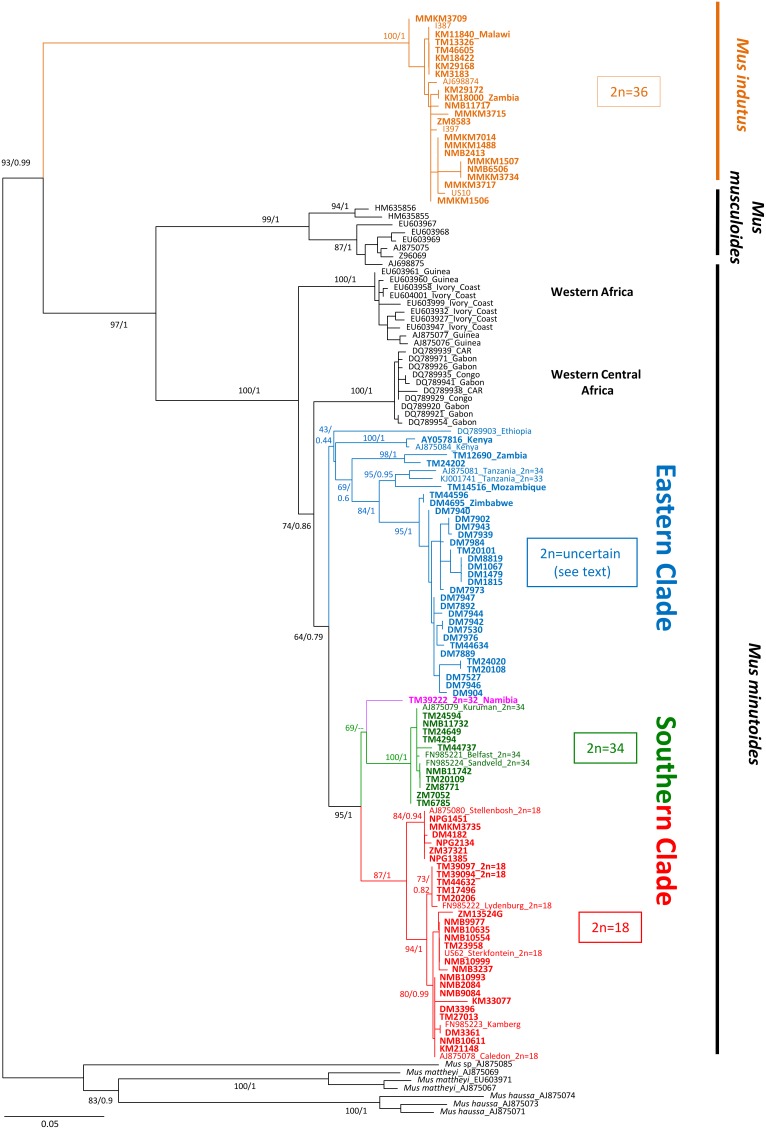
Maximum likelihood tree using cytochrome *b* sequences of the studied samples and those available through GenBank for other specimens and species. Each specimen is designated by its accession or museum number, and country of origin outside of South Africa. Diploid numbers refer to museum or published data. Bootstrap support and the posterior probability values are indicated for the main nodes. The symbol “–” Indicates nodes that were not supported. Colours refer to the different species or cytotypes identified in our study; the museum samples are in bold.

**Figure 4:**
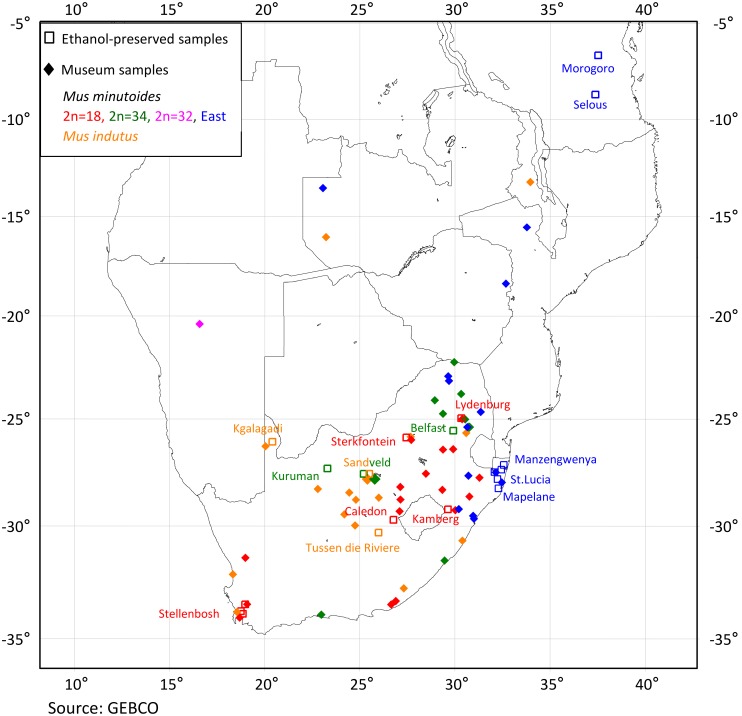
Geographic distribution of the samples. Orange = *Mus indutus*; Red = *M. minutoides* 2n = 18; Green = *M. minutoides* 2n = 34, Pink = *M. minutoides* 2n = 32, Blue = *M. minutoides* Eastern Clade. The open squares refer to previously sampled localities in South Africa.

The ML tree is rooted by *M. haussa*, *M. mattheyi* and *M.* sp_AJ875085. We show that *M. minutoides* and *M. musculoides* are sister species and that *M. indutus* is basal in the tree (Fig. 3). The cytb sequences of the museum samples were clearly partitioned into two different and highly supported species: *M. minutoides* and *M. indutus* (BP = 100, PP = 1). The museum *M. minutoides* samples fell into two major clades (Southern and Eastern), each with several subclades, extending from Southern to Eastern Africa with different but slightly overlapping distributions (Fig. 4).

The southern group is strongly supported (BP = 95, PP = 1) and in turn comprises two subclades that are coincidental with the cytotypes previously found in South African pygmy mice, 2n = 18 and 2n = 34 (respectively red and green in the tree and the map - see Fig. 3 and 4). The 2n = 18 subclade is strongly supported (BP = 87, PP = 1) and includes specimens from the south, central and eastern parts of South Africa. The 2n = 34 subclade is similarly highly supported (BP = 100, PP = 1) and includes three previously karyotyped samples from South Africa (Kuruman, Belfast, Sandveld; 2n = 34; [6], [9]), as well as samples from further North, East and South in South Africa. One specimen from Namibia (TM39222; 2n = 32) shows a weak association with the 2n = 34 South African subclade (ML; BP = 69) and in fact, clusters with the 2n = 18 subclade in Bayesian analyses, although not convincingly (PP = 0.5).

The Eastern group comprises specimens from the north-eastern region of South Africa and extends through Mozambique, Zimbabwe, Zambia, Tanzania, Kenya into Ethiopia. All but one of the South African specimens cluster as a well-supported subclade that forms a sister group to specimens from Tanzania and Mozambique (Fig. 3). Finally, the specimen from Zambia (TM12690), together with a single South African animal (TM24202) occur as an early offshoot of the main Eastern clade with low support (BP = 69, PP = 0.6). The three additional samples from East Africa (Kenya, Ethiopia) split at the base of the Eastern clade but this topology is poorly supported by Bayesian and ML analysis (BP = 43, PP = 0.46). The basal *M. minutoides* clades include specimens from West and West-Central Africa. Interestingly, the mean divergence estimates among samples within the Eastern (2.2%) and Southern (2%) clades are relatively low compared to that between clades (5.8%). These values increase to 5.1%–7.8% in more distant clades (Table S3). It is also noteworthy that the divergence is much lower within *M. indutus* (0.4%) despite a large geographic distribution of the samples (Fig. 4).

## Discussion

### Species Identification and Taxonomy

Our analysis of mitochondrial sequences from museum pygmy mice labelled as *M. minutoides* identified two different species in these collections–*M. indutus* and *M. minutoides*–both of which inhabit Southern Africa [7]. The misidentification of these samples is most likely due to three factors: i) the high level of morphological similarity between the two species [4], ii) specimens were collected outside the currently accepted distribution of *M. indutus* (Fig. 4, [7]) and iii) the formal description of *M. indutus* is more recent than that of *M. minutoides* [21] predating the collection of our oldest samples (Table S1). These incorrect assignments underscore the current difficulties in the morphological diagnosis of species within such a diverse subgenus and stress the need for a careful morphological and morphometrical reappraisal, particularly in the light of recent molecular results [3], [7], [8], [22]. The case of *M. indutus* was further complicated by the co-amplification of nuclear copies of the cytb gene. *Numts* are common in mammals but their number and length present important differences between species [23]. The co-amplification of *numts* is problematic in historic material and requires implementing specific means of detection [14], [24]. This is especially true when the *numts* do not present stop-codon and indels (see for example [25]). The phylogenetic analysis of the *numt* sequences clearly shows that all but two of the sequences cluster together within a single major clade with a very low divergence level. The two “outliers” are more divergent (Fig. 2). The low level of divergence within the major clade may be indicative of a recent common origin for the insertion event, and/or a slower rate of evolution once integration into the nuclear genome occurred (e.g. [26]).

Two other species of pygmy mice have been described in South Africa: *M. orangiae* and *M. neavei* [7]. *Mus orangiae* is restricted to the Free State and Lesotho whereas *M. neavei* has a more extensive distribution from the north of South Africa to south Tanzania [7]. No molecular data are available for these taxa (a recent study morphologically identified two specimens from uMkhuze, Kwazulu-Natal, South Africa as *cf neavei* but these were subsequently non-ambiguously attributed to *M. minutoides* following mitochondrial DNA sequencing [3]). Interestingly, *M. neavei* has, on occasion, been considered a close relative of *M. sorella* [4] for which there is only one sequence available in GenBank. As *M*. *sorella* is distantly related to *M. minutoides* [8], our specific primers might not have been able to amplify cytb of this species and consequently we cannot rule out the presence of *M. neavei* in South Africa.

*Mus orangiae* was listed previously as a subspecies of *M*. *minutoides* [27], [28] and is currently considered a valid species [4], [7], [29]. There are few morphologically diagnostic differences between *M. minutoides* and *M. orangiae* [7], [29], one of these being the number of nipples (10 for *M. minutoides*, 8 for *M. orangiae*). It was not possible to determine the nipple number from the museum study skins. Interestingly, however, this characteristic was not noted in the original and early descriptions of the species [30], [31] and there is no real evidence in the literature to support its reliability as a diagnostic marker. *Mus orangiae* has a very limited distribution in southern Africa with the type locality (Kruisementfontein) located in the north of the Free State Province (South Africa) from which a large number of the museum specimens have originated. This raises the possibility that individuals belonging to one of the two Southern *M*. *minutoides* subclades described herein (Fig. 4) may in fact be attributed to *M. orangiae.* As the type locality of *M. minutoides* is Cape Town [29] and all mice from this subclade have 2n = 18, M. *orangiae* may represent the other Southern subclade (Fig. 4) in which mice have 2n = 34 and are present in the Free State Province. If this is the case, this taxon would best be considered a subspecies of *M*. *minutoides* given the low divergence values within the Southern clade and its phylogenetic position nested within *M. minutoides*. Further molecular studies on museum type specimens of both *M. neavei* and *M. orangiae* are warranted to clarify their systematic status.

### Systematics/Phylogenetic Relationships

The phylogenetic analysis of museum specimens of pygmy mice from Southern Africa suggests an Eastern/Southern partition of the studied samples. The tree retrieved the two previously described subclades from South Africa that are karyotypically differentiated [9]. Further karyotypic subdivision was not detected despite the widespread sampling. Our study did, however, detect the presence of a distinct Eastern African clade that extends southwards into the eastern parts of South Africa (Fig. 4). These results confirm and considerably extend recent data [3]. In summary, therefore, the present and previous reports [2], [3], [8], [22] are in agreement that the South African *M. minutoides* represents an extremely diverse but also a widespread monophyletic complex of pygmy mice that contrasts markedly with the genetic homogeneity of *M. indutus*.

Recently, Lamb et al. [3] have attempted to infer taxonomic status in pygmy mice from sequence divergences. Within the Southern lineage, the three clusters (2n = 18, 2n = 34, Namibia: 2n = 32) differ by less than 3.8% average sequence divergence (range: 2.5–3.8%). Moreover, the ranges of the 2n = 18 and 2n = 34 cytotypes overlap in South Africa and in fact one F1 hybrid female has been described on chromosomal grounds (2n = 26; [9]). These observations are suggestive of a subspecific relationship among clades–certainly so among the two South African chromosomal groups. By comparison, the Eastern clade appears more homogeneous (average divergence: 2.2%), and is separated from the Southern clades by 5.4%–6.6% sequence divergence. Claims of species status for pygmy mice belonging to the Eastern clade [3], distinct from those in the Southern clade, find support from both the level of divergence and their occurrence in sympatry in Botswana and Namibia [3], [32]. However, the interclade relationships, as well as some of the sister group relationships within the clades, remain poorly resolved and additional molecular analyses (nuclear sequences) and further sampling from these regions are clearly required.

### Geographic Distribution

The molecular analysis of museum specimens has provided considerable additional information on the distribution of *M. indutus* as well as of the different lineages of *M. minutoides* in Southern Africa (Fig. 4). The Southern 2n = 18 cytotype is evidently endemic to South Africa whereas the 2n = 34 subclade occurs in all of South Africa except the western region and extends further north into Namibia, Botswana and Angola [3]. The Eastern clade is present in the eastern part of South Africa as well as Swaziland and Zimbabwe; it (or a closely related form) extends north through Mozambique, Namibia, Botswana, Tanzania to Kenya and possibly Ethiopia [3], [32]. It is particularly interesting that all three groups overlap in the northeastern region of South Africa, marking this area as a focus for further investigations.

Based on the samples analysed herein, our assessment of the limits of *M. indutus* suggests that it is far more widely distributed than current information would suggest. Given the assumption that the so-called “Desert Pygmy Mouse” species inhabits semiarid environments, its presence in the extreme south of the Western Cape (Fig. 4) and at several localities along the east coast and in the northern part of South Africa, as well as Malawi is surprising (Fig. 4) [7], [33]. However, adjustments to the species’ range suggested by our inclusion of museums specimens should be carefully considered since their provenance may be suspect in some instances (for example, Table Mountain is not in an arid region of the western Cape suggesting that the *indutus* sample purportedly collected there in 1903–04 is incorrect; Fig. 4, Table S1). Additional samples are needed to confirm the presence of *M. indutus* in these questionable areas.

### Chromosomal Inference

Previous studies of pygmy mice from South Africa revealed perfect congruence between the mitochondrial-based phylogeny and karyotypes [9]. This pattern is recovered in the present analysis as the Southern clade consists of two distinct groups, each of which is characterised by a different diploid number (2n = 18 vs 2n = 34). The consistency between the cytb sequence tree and karyotypes suggests that a tentative chromosomal assignment of the museum specimens from these clades may be inferred. This is supported by the karyotypes of two of the museum specimens (TM39094, TM39097; 2n = 18). It is noteworthy that although previous chromosomal analyses have shown that the 2n = 18 karyotype was not homogeneous and in fact harboured four distinct groups (all with 18 chromosomes, but with different combinations of centric fusions [9]), only one of these subgroups is strongly supported in our tree (Fig. 3) and includes the karyotyped specimen from Stellenbosch. It seems reasonable to infer therefore, that the six individuals that cluster within this clade most probably share an identical 2n = 18 karyotype. This suggests the delimitation of this cytotype to the southernmost region of South Africa (all localities around Stellenbosch and one North of it; see Fig. 4). Karyotypic inference for the Southern 2n = 34 subclade is less straightforward as the specimen from Namibia has 2n = 32 indicating the presence of an additional rearrangement. The nature and extent of chromosome variability within this subclade should be assessed by standard cytogenetic methods.

The Eastern clade consists of five groups among which chromosomal data are available for one of them (Tanzania, 2n = 33 and 34). Whereas previously studied specimens from Zambia carried the widespread diagnostic sex autosome fusions X.1 and Y.1 [34], a recent cytogenetic study [35] reports the unexpected absence of the X.1 fusion in *M. minutoides* from Tanzania and South Africa suggesting a derived (secondary loss) chromosomal complement for these mice. The highly supported subclade grouping the Tanzanian pygmy mice with the newly described South African lineage (BP = 84; PP = 1; Fig. 3) suggests that the cytogenetic analysis of specimens from Mozambique and Zimbabwe is a priority. Clarification of the presence/absence of the X.1 fusion in the entire Eastern clade will determine the geographical occurrence and the chronological history of this rearrangement.

## Conclusion

The analysis of museum samples has significantly expanded what is known of the systematics of *Nannomys* species in Southern Africa. It has shown that good quality data can be obtained from relatively old museum specimens (collected during the first half of the 20th century) irrespective of idiosyncrasies in the preservation of study skins and skulls. The approach holds promise for taxonomic and phylogeographic surveys of taxa that are rare or difficult to sample, as well as for monitoring biodiversity changes through time. Given the extent of morphological uniformity encountered in *Nannomys* [4], [5], further research involving Museum Type Specimens (for which there are currently no molecular data) will more firmly anchor the phylogeny of this taxonomically challenging complex of mice.

## Supporting Information

Figure S1 **Details of the processing of the museum tissue snips.** The absence of amplification is shown in white and contaminated samples in grey. The sequences corresponding to *M. minutoides* are presented in black and those of *M. indutus* in orange. The samples are classified according to their age: >50: samples collected before 1960, <50: after 1960. Y-axis gives the number of animals analyzed from each museum sampled.
(PDF)

Figure S2 **Alignments of the** ***M. indutus*** **cloned sequences compared to sequences of different species of** ***Nannomys.*** **a) nucleotide alignment, b) protein alignment.** For each sequence the number of clones is indicated. The mitochondrial sequences are indicated by the orange background, the *numts* are in the orange rectangle. The arrows point to the aminoacid substitutions (red) and stop-codons (black) characteristic of the numt sequences, ? indicates a position that was not sequenced, N represents any nucleotide, and X any amino-acid.
(PDF)

Table S1 List of the museum (skin and skull) and ethanol-preserved samples (tissue) sequenced in this study. For each, we include information taken from specimen labels: Museum accession number, date of collection, sex, species designation and collection locality where available.
(XLSX)

Table S2 List of the Genbank sequences included in the phylogenetic analysis.
(DOCX)

Table S3 K2P genetic distances (%) between (below diagonal) and within (diagonal) the different lineages present in our study.
(DOCX)

File S1 **Cytb sequence data in fasta format.**
(TXT)
