# Supplementary figures and images for: A Phylogeographic Survey of the Pygmy Mouse Mus minutoides in South Africa: Taxonomic and Karyotypic Inference from Cytochrome b Sequences of Museum Specimens
Source: PLoS One. 2014 Jun 6;9(6):e98499. doi: 10.1371/journal.pone.0098499 (PMC4048158; doi:10.1371/journal.pone.0098499)

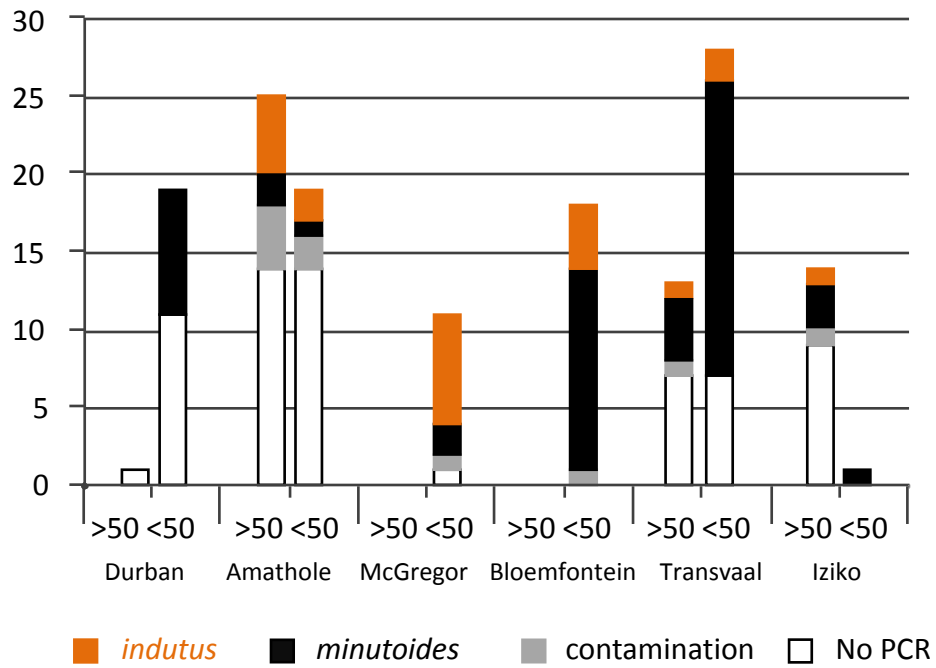

Supplement: Figure S1 — Details of the processing of the museum tissue snips. The absence of amplification is shown in white and contaminated samples in grey. The sequences corresponding to M. minutoides are presented in black and those of M. indutus in orange. The samples are classified according to their age: >50: samples collected before 1960, <50: after 1960. Y-axis gives the number of animals analyzed from each museum sampled. (PDF) [file pone.0098499.s001.pdf]
