# Supplementary material for: A Phylogeographic Survey of the Pygmy Mouse Mus minutoides in South Africa: Taxonomic and Karyotypic Inference from Cytochrome b Sequences of Museum Specimens
Source: PLoS One. 2014 Jun 6;9(6):e98499. doi: 10.1371/journal.pone.0098499 (PMC4048158; doi:10.1371/journal.pone.0098499)
Supplement: Figure S2 — Alignments of the M. indutus cloned sequences compared to sequences of different species of Nannomys. a) nucleotide alignment, b) protein alignment. For each sequence the number of clones is indicated. The mitochondrial sequences are indicated by the orange background, the numts are in the orange rectangle. The arrows point to the aminoacid substitutions (red) and stop-codons (black) characteristic of the numt sequences, ? indicates a position that was not sequenced, N represents any nucleotide, and X any amino-acid. (PDF) [file pone.0098499.s002.pdf]

A

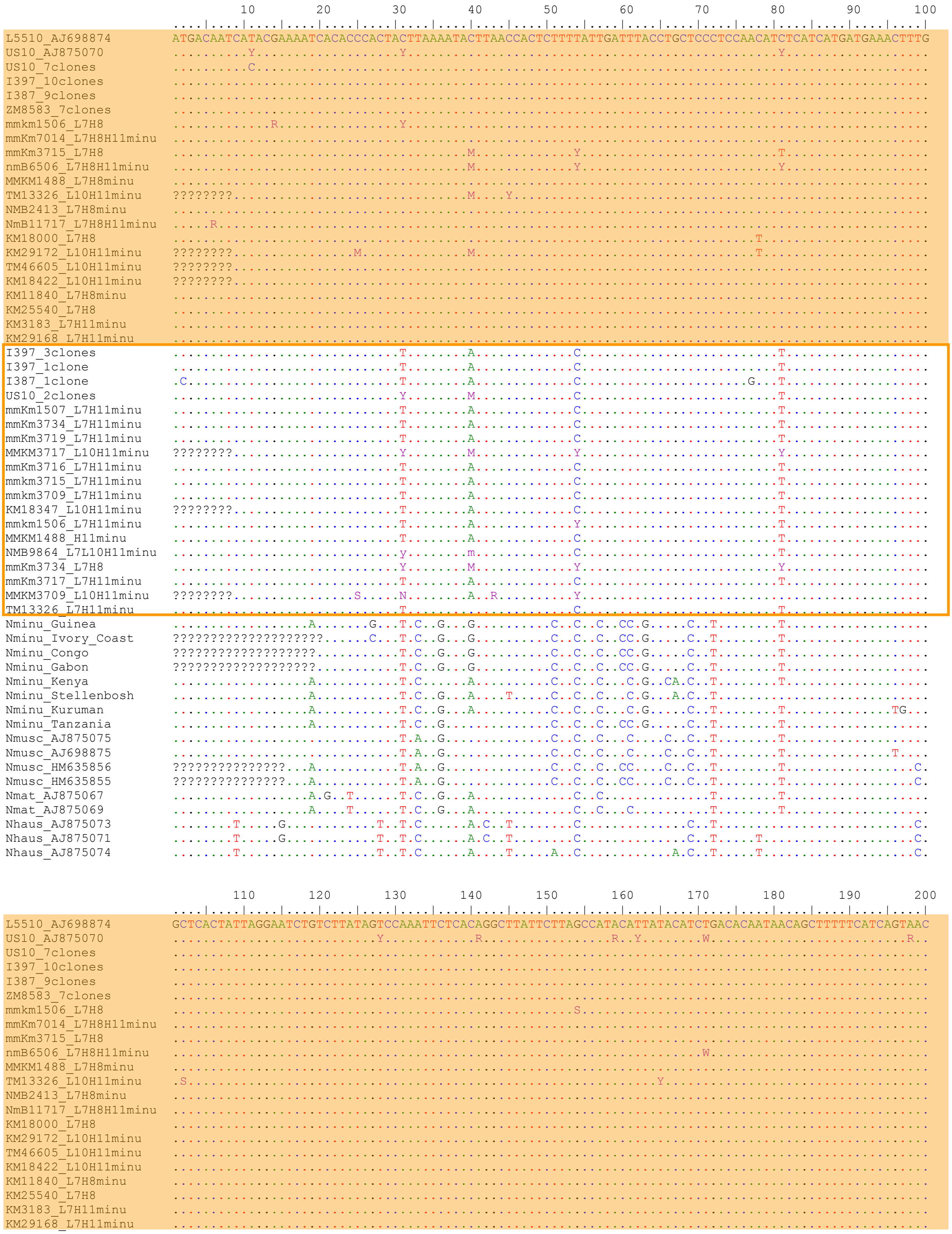
10 20 30 40 50 60 70 80 90 100
L5510_AJ698874
US10_AJ875070
US10_7clones
I397_10clones
I387_9clones
ZM8583_7clones
mmkm1506_L7H8
mmKm7014_L7H8H11minu
mmKm3715_L7H8
nmB6506_L7H8H11minu
MMKM1488_L7H8minu
TM13326_L10H11minu
NMB2413_L7H8minu
NmB11717_L7H8H11minu
KM18000_L7H8
KM29172_L10H11minu
TM46605_L10H11minu
KM18422_L10H11minu
KM11840_L7H8minu
KM25540_L7H8
KM3183_L7H11minu
KM29168_L7H11minu
I397_3clones
I397_1clone
I387_1clone
US10_2clones
mmKm1507_L7H11minu
mmKm3734_L7H11minu
mmKm3719_L7H11minu
MMKM3717_L10H11minu
mmKm3716_L7H11minu
mmKm3715_L7H11minu
mmkm3709_L7H11minu
KM18347_L10H11minu
mmkm1506_L7H11minu
MMKM1488_H11minu
NMB9864_L7L10H11minu
mmKm3734_L7H8
mmKm3717_L7H11minu
MMKM3709_L10H11minu
TM13326_L7H11minu
Nminu_Guinea
Nminu_Ivory_Coast
Nminu_Congo
Nminu_Gabon
Nminu_Kenya
Nminu_Stellenbosh
Nminu_Kuruman
Nminu_Tanzania
Nmusc_AJ875075
Nmusc_AJ698875
Nmusc_HM635856
Nmusc_HM635855
Nmat_AJ875067
Nmat_AJ875069
Nhaus_AJ875073
Nhaus_AJ875071
Nhaus_AJ875074
110 120 130 140 150 160 170 180 190 200
L5510_AJ698874
US10_AJ875070
US10_7clones
I397_10clones
I387_9clones
ZM8583_7clones
mmkm1506_L7H8
mmKm7014_L7H8H11minu
mmKm3715_L7H8
nmB6506_L7H8H11minu
MMKM1488_L7H8minu
TM13326_L10H11minu
NMB2413_L7H8minu
NmB11717_L7H8H11minu
KM18000_L7H8
KM29172_L10H11minu
TM46605_L10H11minu
KM18422_L10H11minu
KM11840_L7H8minu
KM25540_L7H8
KM3183_L7H11minu
KM29168_L7H11minu

```
I397_3clones              ........................................G.................G..C........A.................C........G..
I397_1clone               ........................................G.................G..C........A.................C...........
I387_1clone               ........................................G.................G..C........A.................C........G..
US10_2clones              ........................................R.................R..C........A.................Y........R..
mmKm1507_L7H11minu        ........................................G.................G..C........A.................C........G..
mmKm3734_L7H11minu        ........................................G.................G..C........A.................C........G..
mmKm3719_L7H11minu        ........................................G.................G..C........A.................C........G..
MMKM3717_L10H11minu       ........................................R.................R...........W..........................R..
mmKm3716_L7H11minu        ........................................G.................G..C........A.................C........G..
mmkm3715_L7H11minu        ........................................G.................G..C........A.................C........G..
mmkm3709_L7H11minu        ........................................G.................G..C........A.................C........G..
KM18347_L10H11minu        ........................................G.................G..C........A.................C........G..
mmkm1506_L7H11minu        ........................................R.................R..Y........A.................Y........R..
MMKM1488_H11minu          ........................................G.................G..C........A.................C........G..
NMB9864_L7L10H11minu      ........................................r.................r..C........A.................C........G..
mmKm3734_L7H8             ........................................R.................R..Y........W.................Y........R..
mmKm3717_L7H11minu        ........................................G.................G..C........A.................C........G..
MMKM3709_L10H11minu       ..........................................................W.........................................
TM13326_L7H11minu         ..................................CA......................G..C........A..........................G..
Nminu_Guinea              .A..T..TC.......T..C.....T........C..T............C....A.....C...T....A..............C..C...........
Nminu_Ivory_Coast         .A..T..TC.......T..C.....T........C..T.....T......C....A.....C..C.....A..............C..C...........
Nminu_Congo               .A..T...C..........C..............C.........C.....C....A.....C........A.......N.N....C..C........T..
Nminu_Gabon               .A..T...C..........C......A.......C.........C.....C....A.....C........A..............C..C........T..
Nminu_Kenya               .A..T..............C........T.....C.........C.....C....A.....C........A..............C..C...........
Nminu_Stellenbosh         .A..T..............C..............C.........C.....C....A.....C..C.....A.....G........C..C...........
Nminu_Kuruman             .A..T.........G....C....C.........C.........C.....C....A.....C..C.....A.....G........C..C...........
Nminu_Tanzania            .A..T..............C..............C...............C....A.....C..C.....A..............C..C...........
Nmusc_AJ875075            .G..T..GC..........C.A...........C.........C..........T.....C..C.....A..............C..C...........
Nmusc_AJ698875            .G..T..GC..........C.A...........C.........C..........T.....C..C.....A..............C..C...........
Nmusc_HM635856            .G..T..GC..........C.A...........C.....N...C..........T.....C..C.....A..............C..C...........
Nmusc_HM635855            .G..T...C..........C.A...........C.....N...C..........T.....C..C.....A..............C..C...........
Nmat_AJ875067             .......CC....G.T..C..C...A.....G...........C..........A..............A.....T.C......C..C........C..
Nmat_AJ875069             .G.....CC..........C.A.........G...........C..........A..............A.....T.C......C..C........T..
Nhaus_AJ875073            .A.....CC.......T.................C..............TC....A..............A..............C..C..G........
Nhaus_AJ875071            .A.....CC.......T.................C.........C....TC....A........C.....A..............C..C..G........
Nhaus_AJ875074            .A.....CC.......T..C..............C..............TC....A........C.....A..............C..C..G........

                                   210       220       230       240       250       260       270       280       290       300
                          ....|....|....|....|....|....|....|....|....|....|....|....|....|....|....|....|....|....|....|....|
L5510_AJ698874            ACATATCTGTCGAGACGTAAACTACGGATGATTAATCCGATACATACACGCAAACGGAGCCTCCATATTCTTTATCTGCTTATTTATACACGTAGGACGA
US10_AJ875070             ...Y......................................T.....T...................................................
US10_7clones              ..........................................T.....T...................................................
I397_10clones             ..........................................T.........................................................
I387_9clones              ..........................................T.........................................................
ZM8583_7clones            ..........................................T.........................................................
mmkm1506_L7H8             ..........................................T.........................................................
mmKm7014_L7H8H11minu      ..........................................T.....T...................................................
mmKm3715_L7H8             ..........................................T.........................................................
nmB6506_L7H8H11minu       ..........................................T.....T...................................................
MMKM1488_L7H8minu         ..........................................T.....T...................................................
TM13326_L10H11minu        ..........................................T.........................................................
NMB2413_L7H8minu          ..........................................T.....T...................................................
NmB11717_L7H8H11minu      ..........................................T.........................................................
KM18000_L7H8              ..........................................T.........................................................
KM29172_L10H11minu        ........................M.................T.........................................................
TM46605_L10H11minu        ..........................................T.........................................................
KM18422_L10H11minu        ..........................................T.........................................................
KM11840_L7H8minu          ..........................................T.........................................................
KM25540_L7H8              ..........................................T.........................................................
KM3183_L7H11minu          ..........................................T.........................................................
KM29168_L7H11minu         ..........................................T.........................................................
I397_3clones              ...C.......A..............................T.....T....................................T...........T..
I397_1clone               T........C.....T........T......C......A...T....G......T..............T.....T..TC.....C.G..T.......A.
I387_1clone               ...C.......A..............................T.....T....................................T...........T..
US10_2clones              ...Y.......R..................R...........T.....T......K.............................T...........Y..
mmKm1507_L7H11minu        ...C.......A..............................T.....T....................................T...........T..
mmKm3734_L7H11minu        ...C.......A..............................T.....T....................................T...........T..
mmKm3719_L7H11minu        ...C.......A..............................T.....T....................................T...........T..
MMKM3717_L10H11minu       ...Y.......R..............................T.....T....................................W...........Y..
mmKm3716_L7H11minu        ...C.......A..............................T.....T....................................T...........T..
mmkm3715_L7H11minu        ...C.......A..............................T.....T....................................T...........T..
mmkm3709_L7H11minu        ...C.......A..............................T.....T....................................T...........T..
KM18347_L10H11minu        ...C.......A..............................T.....T....................................T...........T..
mmkm1506_L7H11minu        ...Y.......R..............................T.....T....................................W...........Y..
MMKM1488_H11minu          ...C.......A..............................T.....T.............................Y......T...........T..
NMB9864_L7L10H11minu      ...y.......r..............................T.....T....................................T...........T..
mmKm3734_L7H8             ...Y.......R..............................T.....T....................................W..............
mmKm3717_L7H11minu        ...C.......A..............................T.....T....................................T...........T..
MMKM3709_L10H11minu       .........C................................T.........................................................
TM13326_L7H11minu         ..............................G.......T...T.....T....................................T..............
Nminu_Guinea              .........C..............T......C..........TT....T..C...........T..G..................T.............G
Nminu_Ivory_Coast         .........C..............T......C..........TT....T..C...........T.....................T.............G
Nminu_Congo               ...C.....C.....T........T......C...........T....T..C...........T.....................T....T........G
Nminu_Gabon               ...C.....C.....T........T......C...........T....T..C...........T.....................T....T........G
Nminu_Kenya               ...C.....C.....T........T......C...........T....T..C...........T.....................T.............G
Nminu_Stellenbosh         .........C.....T........T.....GC...........C....T..C.....G.....T..............T......T.............G
Nminu_Kuruman             ......T..C.....T........T.....GC...........C....T..C.....G.....T.....................C.............G
```

```
Nminu_Tanzania          ...............T........T......C...........C....T..C...........T..............T......T.............G
Nmusc_AJ875075          .........C.....T........T.................TT.......C...........................C....CT.............G
Nmusc_AJ698875          .........C.....T........T.................TT.......C................................CT.............G
Nmusc_HM635856          .........C.....T........N.................TT.......C.................................T.............G
Nmusc_HM635855          .........C.....T........N..GN.............TT.......C.........N..............N........T...........N.G
Nmat_AJ875067           C..............T...............C..........T.....T.....T........T.....T...............C.G..T.........
Nmat_AJ875069           C..............T...............C................T..............T.....................C.G..T.........
Nhaus_AJ875073          C........C.....T..T..T....................T.........................................CC.G...A........
Nhaus_AJ875071          C........C.....T..T............C.....................................................C.G............
Nhaus_AJ875074          C........C.....T..T.......................T..........................................C.G........G...

                                 310       320       330       340       350       360       370       380       390       400
                        ....|....|....|....|....|....|....|....|....|....|....|....|....|....|....|....|....|....|....|....|
L5510_AJ698874          GGAATATACTATGGATCTTACACATTTATAGAAACATGAAATATTGGGGTTATTCTATTATTCGCTGTGATAGCCACAGCATTCATAGGTTATGTCCTCC
US10_AJ875070           .................Y..................................................................................
US10_7clones            ....................................................................................................
I397_10clones           ....................................................................................................
I387_9clones            ...................................................................................T................
ZM8583_7clones          ....................................................................................................
mmkm1506_L7H8           ....................................................................................................
mmKm7014_L7H8H11minu    ....................................................................................................
mmKm3715_L7H8           .................................................................................................A..
nmB6506_L7H8H11minu     ..............................................................Y.....................................
MMKM1488_L7H8minu       ....................................................................................................
TM13326_L10H11minu      .....R.............................................................................T................
NMB2413_L7H8minu        ....................................................................................................
NmB11717_L7H8H11minu    ..............G.........................................................K...........................
KM18000_L7H8            ....................................................................................................
KM29172_L10H11minu      ....................................................................................................
TM46605_L10H11minu      ...................................................................................T................
KM18422_L10H11minu      ...................................................................................T................
KM11840_L7H8minu        ...................................................................................T................
KM25540_L7H8            ...................................................................................T................
KM3183_L7H11minu        ...................................................................................T................
KM29168_L7H11minu       ...................................................................................T................
I397_3clones            .................C............................................T.....A...............................
I397_1clone             ...G.G........G..C..T................TGG.GTAATTCTACTA.T.GC.G.AATAGCCACATCATTT.TAGG.T..GTTC.TCCATGAGG
I387_1clone             .................C............................................T.....A.....................C.........
US10_2clones            .................C............................................T.....A...............................
mmKm1507_L7H11minu      .................C............................................T.....A...............................
mmKm3734_L7H11minu      .................C............................................T.....A...............................
mmKm3719_L7H11minu      .................C............................................T.....A...............................
MMKM3717_L10H11minu     ..............................................................Y.....................................
mmKm3716_L7H11minu      .................C............................................T.....A...............................
mmkm3715_L7H11minu      .................C............................................T.....A...............................
mmkm3709_L7H11minu      .................C............................................T.....A...............................
KM18347_L10H11minu      .................C............................................T.....A...............................
mmkm1506_L7H11minu      .................Y........Y...................................Y.....R...............................
MMKM1488_H11minu        .................C............................................T.....A...............................
NMB9864_L7L10H11minu    .................C............................................T.....A...............................
mmKm3734_L7H8           .................Y..................................................................................
mmKm3717_L7H11minu      .................C............................................T.....A...............................
MMKM3709_L10H11minu     ....................................................................................................
TM13326_L7H11minu       .................C............................................T.....A...............................
Nminu_Guinea            .....G.....C.....C................................C..C...C....T..A.................T................
Nminu_Ivory_Coast       .....G.....C.....C................................C..C...C....T..A.................T................
Nminu_Congo             ...........C.....C....................................T..C.G..T..A.................T...........T....
Nminu_Gabon             ...........C.....C....................................T..C.G..T..A.................T...........T....
Nminu_Kenya             .....G...........C...................................CT..C.G..T..A.................T..G.............
Nminu_Stellenbosh       .....G.....C.....C....................................T..C....T..A..A..............T................
Nminu_Kuruman           ..G..G.....C.....C....................................T..C.G..T..A..C..............T..G.............
Nminu_Tanzania          .....G.....C.....C..T.......................C........CT..C....T..A.................T.....C..........
Nmusc_AJ875075          ..G..G.....C........T.....C....................A..............T..A..A.........T....T...........T..T.
Nmusc_AJ698875          ..G..G.....C..............C....................A..A...........T..A..A.........T....T...........T..T.
Nmusc_HM635856          .....G................................G........A..A...........T..A..T..............T..............A.
Nmusc_HM635855          ..G..G.....N................................N..A..A...........T..A..T..............T...........T..A.
Nmat_AJ875067           ........T........C..T..........................A..A...T..C....T..A..A.....T........T...........A..T.
Nmat_AJ875069           ........T..C........T..........................A.....CT..C....T..A..A.....T........T...........T..T.
Nhaus_AJ875073          .................C..T..........................A..AG.C..CC....T..A..A.....T...T....T...........T....
Nhaus_AJ875071          .................C..T..........................A..AG.C..CC....T..A..A.....T...T....T...........A....
Nhaus_AJ875074          .....G..............T..........................A..AG.C..CC....T..A..A.....T...T................A....

                                 410       420       430       440       450       460
                        ....|....|....|....|....|....|....|....|....|....|....|....|....
L5510_AJ698874          CATGAGGACAAATATCATTTTGAGGGGCCACAGTAATTACAAATCTTCTATCAGCAGTTCCATA
US10_AJ875070           ......................R...................................Y.....
US10_7clones            ................................................................
I397_10clones           ........................................................A.......
I387_9clones            ................................................................
ZM8583_7clones          ..
mmkm1506_L7H8           ...
mmKm7014_L7H8H11minu    ................................................................
mmKm3715_L7H8           .R.
nmB6506_L7H8H11minu     ........................A...............................
MMKM1488_L7H8minu       ...
TM13326_L10H11minu      ...............................................................
```

```
NMB2413_L7H8minu       ...
NmB11717_L7H8H11minu   ..........................................................
KM18000_L7H8           ...
KM29172_L10H11minu     ................................................................
TM46605_L10H11minu     ................................................................
KM18422_L10H11minu     ................................................................
KM11840_L7H8minu       ...
KM25540_L7H8           ...................C.....
KM3183_L7H11minu       ................................................................
KM29168_L7H11minu      ................................................................
I397_3clones           ......................G.A...............................A.C.....
I397_1clone            ACAA.T.T..TT.TGAGGAGCA.CA.TAAT..CA...CTATT...AG....TTCATA
I387_1clone            ......................G.A...............................A.C.....
US10_2clones           ......................G.R...............................R.C.....
mmKm1507_L7H11minu     ......................G.A...............................A.C.....
mmKm3734_L7H11minu     ......................G.A...............................A.C.....
mmKm3719_L7H11minu     ......................G.A...............................A.C.....
MMKM3717_L10H11minu    ......................R.........................................
mmKm3716_L7H11minu     ......................G.A...............................A.C.....
mmkm3715_L7H11minu     ......................G.A...............................A.C.....
mmkm3709_L7H11minu     ......................G.A...............................A.C.....
KM18347_L10H11minu     ......................G.A...............................A.C.....
mmkm1506_L7H11minu     ......................R.A...............................A.C.....
MMKM1488_H11minu       ......................G.A.............
NMB9864_L7L10H11minu   ........................A...............................A.C.....
mmKm3734_L7H8          ...
mmKm3717_L7H11minu     ......................G.A...............................A.C.....
MMKM3709_L10H11minu    ................................................................
TM13326_L7H11minu      ......................G.....................
Nminu_Guinea           ....G..G..G.................T..............C...........CA.C.....
Nminu_Ivory_Coast      ....G..G..G.................T..............C...........CA.C.....
Nminu_Congo            ....G..G..G..............A..T..............C...........CA.......
Nminu_Gabon            ....G..G..G..............A..T..............C...........CA.......
Nminu_Kenya            ....G..G..G.................T..............C...........CA.......
Nminu_Stellenbosh      ....G.....G.................T..............C...........CA.......
Nminu_Kuruman          ....G..G..G.................T.....G........C...........CA.C.....
Nminu_Tanzania         ..........G.................T........C.....C...........TA.......
Nmusc_AJ875075         .........................A..T........C.....C..CT.......CA.......
Nmusc_AJ698875         .........................A..T........C.....C...........CA.......
Nmusc_HM635856         .........................A...........C.....C.........N.CA.......
Nmusc_HM635855         .........................A...........C.....C...........CA.......
Nmat_AJ875067          ...................C.....T.....C.......................TA.......
Nmat_AJ875069          ...................C.....T.....C.......................TA.......
Nhaus_AJ875073         ...............................C.......................TA.C.....
Nhaus_AJ875071         ...............................C..G....................TA.C.....
Nhaus_AJ875074         ...............................C...............T.......TA.C.....
```

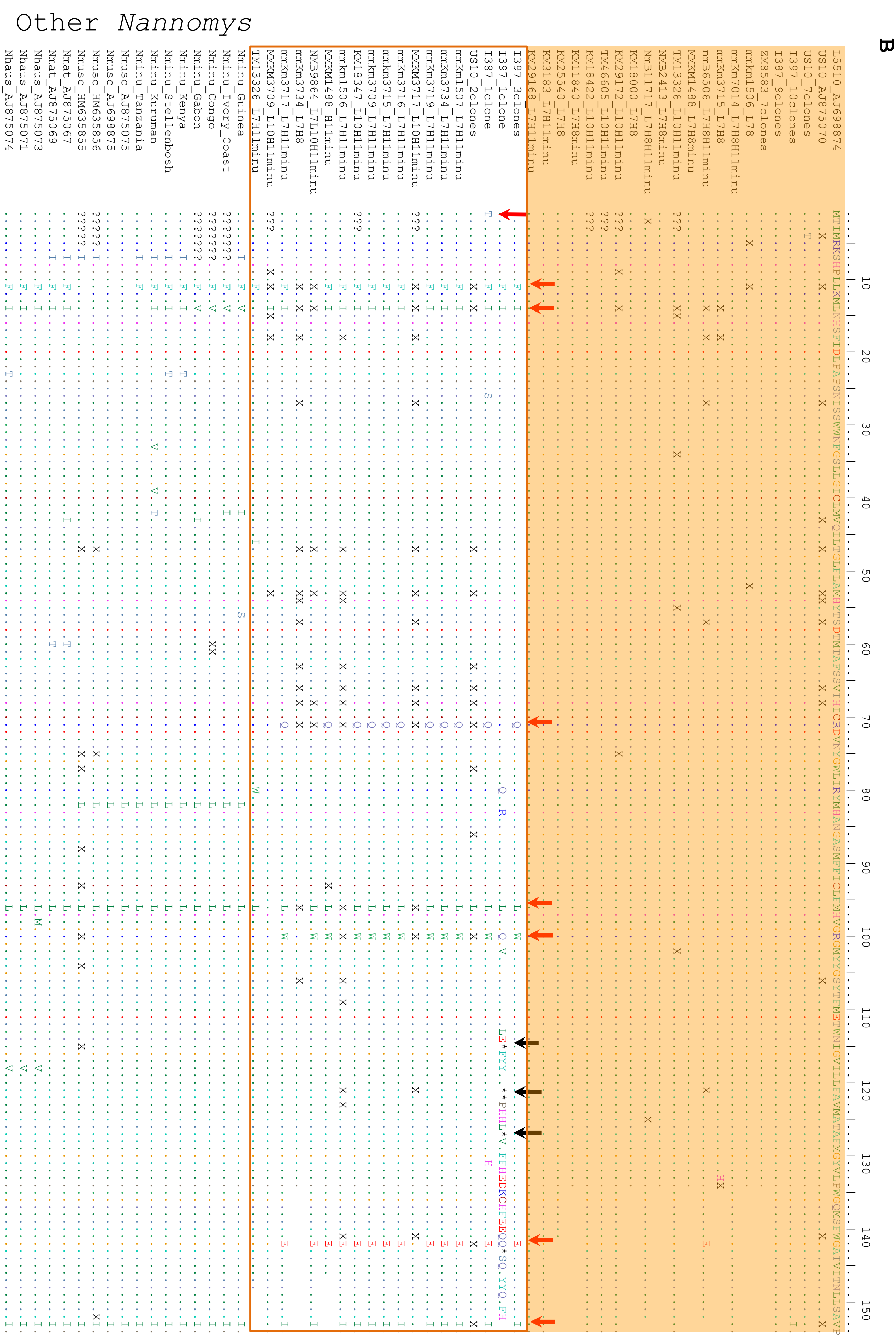
B
Other Nannomys
MTIMRKSHPLLKMLNHSFIDLPAPSNISSWWNFGSLLGICLMVQILTGLFLAMHYTSDTMTAFSSVTHICRDVNYGWLIRYMHANGASMFFICLFMHVGRGMYYGSYTFMETWNIGVILLFAVMATAFMGYVLPWGQMSFWGATVITNLLSAVP
10 20 30 40 50 60 70 80 90 100 110 120 130 140 150
L5510_AJ698874
US10_AJ875070
US10_7clones
I397_10clones
I387_9clones
ZM8583_7clones
mmKm1506_L78
mmKm7014_L7H8H11minu
mmKm3715_L7H8
mmB6506_L7H8H11minu
MMKM1488_L7H8minu
TM13326_L10H11minu
NMB2413_L7H8minu
NmB11717_L7H8H11minu
KM18000_L7H8
KM29172_L10H11minu
TM46605_L10H11minu
KM18422_L10H11minu
KM11840_L7H8minu
KM25540_L7H8
KM3183_L7H11minu
KM29168_L7H11minu
I397_3clones
I397_1clone
I387_1clone
US10_2clones
mmKm1507_L7H11minu
mmKm3734_L7H11minu
mmKm3719_L7H11minu
MMKM3717_L10H11minu
mmKm3716_L7H11minu
mmKm3715_L7H11minu
mmKm3709_L7H11minu
KM18347_L10H11minu
mmKm1506_L7H11minu
MMKM1488_H11minu
NMB9864_L7L10H11minu
mmKm3734_L7H8
mmKm3717_L7H11minu
MMKM3709_L10H11minu
TM13326_L7H11minu
Nminu_Guinea
Nminu_Ivory_Coast
Nminu_Congo
Nminu_Gabon
Nminu_Kenya
Nminu_Stellenbosh
Nminu_Kuruman
Nminu_Tanzania
Nmusc_AJ875075
Nmusc_AJ698875
Nmusc_HM635856
Nmusc_HM635855
Nmat_AJ875067
Nmat_AJ875069
Nhaus_AJ875073
Nhaus_AJ875071
Nhaus_AJ875074
