# Supplementary material for: A Phylogeographic Survey of the Pygmy Mouse Mus minutoides in South Africa: Taxonomic and Karyotypic Inference from Cytochrome b Sequences of Museum Specimens
Source: PLoS One. 2014 Jun 6;9(6):e98499. doi: 10.1371/journal.pone.0098499 (PMC4048158; doi:10.1371/journal.pone.0098499)
Supplement: Table S2 — List of the Genbank sequences included in the phylogenetic analysis. (DOCX) [file pone.0098499.s004.docx]

| Species | Sub-group/cytotype | Accessions |
| --- | --- | --- |
| *Mus minutoides* | Southern Africa / 2n=18 | FN985223, AJ875080, AJ875078, FN985222 |
| *Mus minutoides* | Southern Africa / 2n=34 | AJ875079, FN985224, FN985221 |
| *Mus minutoides* | Eastern | AJ875081, KJ001741 |
| *Mus minutoides* | Eastern + Southern Africa | AJ875084, AY057816, DQ789903 |
| *Mus minutoides* | Western Central Africa | DQ789971, DQ789954, DQ789941, DQ789939, DQ789938, DQ789935, DQ789929, DQ789926, DQ789921, DQ789920 |
| *Mus minutoides* | Western Africa | AJ875076, AJ875077, EU603961, EU603960, EU603958, EU603947, EU603932, EU603927, EU604001, EU603999 |
| *Mus musculoides* |  | AJ875075, Z96069, AJ698875, EU603969, EU603968, EU603967, HM635856, HM635855 |
| *Mus indutus* |  | AJ875070, AJ698874 |
| *Mus mattheyi* |  | AJ875067, AJ875069, EU603971 |
| *Mus haussa* |  | AJ875073, AJ875071, AJ875074 |
| *Mus sp.* |  | AJ875085 |
