# Supplementary material for: A Phylogeographic Survey of the Pygmy Mouse Mus minutoides in South Africa: Taxonomic and Karyotypic Inference from Cytochrome b Sequences of Museum Specimens
Source: PLoS One. 2014 Jun 6;9(6):e98499. doi: 10.1371/journal.pone.0098499 (PMC4048158; doi:10.1371/journal.pone.0098499)
Supplement: Table S3 — K2P genetic distances (%) between (below diagonal) and within (diagonal) the different lineages present in our study. (DOCX) [file pone.0098499.s005.docx]

|  | Lineages | 1 | 2 | 3 | 4 | 5 | 6 | 7 | 8 | 9 | |
| --- | --- | --- | --- | --- | --- | --- | --- | --- | --- | --- | --- |
| 1 | 2n=18 | 0.7 |  |  |  |  |  |  |  |  |  |
| 2 | 2n=32 | 2.5 | na |  |  |  |  |  |  |  |  |
| 3 | 2n=34 | 3.8 | 3.1 | 0.3 |  |  |  |  |  |  |  |
| 4 | Southern | na | na | na | 2.0 |  |  |  |  |  |  |
| 5 | Eastern | 5.5 | 5.4 | 6.6 | 5.8 | 2.2 |  |  |  |  |  |
| 6 | West Central | 5.3 | 4.9 | 6.3 | 5.6 | 5.1 | 0.5 |  |  |  |  |
| 7 | Western | 6.4 | 6.6 | 7.8 | 6.8 | 6.3 | 5.1 | 0.9 |  |  |  |
| 8 | *M. musculoides* | 8.9 | 9.7 | 10.8 | 9.4 | 9.2 | 8.8 | 9.4 | 2.2 |  |  |
| 9 | *M. indutus* | 13.1 | 14.5 | 15.6 | 13.9 | 13.4 | 13.8 | 13.5 | 12.8 | 0.4 |  |

na = not available
